# Development of muscle ultrasound density in healthy fetuses and infants

**DOI:** 10.1371/journal.pone.0235836

**Published:** 2020-07-10

**Authors:** Renate J. Verbeek, Petra B. Mulder, Krystyna M. Sollie, Johannes H. van der Hoeven, Wilfred F. A. den Dunnen, Natalia M. Maurits, Deborah A. Sival

**Affiliations:** 1 Department of (Pediatric) Neurology, University Medical Center Groningen, University of Groningen, Groningen, The Netherlands; 2 Department of Obstetrics, University Medical Center Groningen, University of Groningen, Groningen, The Netherlands; 3 Department of Pathology, University Medical Center Groningen, University of Groningen, Groningen, The Netherlands; 4 Department of Pediatrics, Beatrix Children’s Hospital, University Medical Center Groningen, University of Groningen, The Netherlands; INIA, SPAIN

## Abstract

Muscle ultrasound density (MUD) is a non-invasive parameter to indicate neuromuscular integrity in both children and adults. In healthy fetuses and infants, physiologic MUD values during development are still lacking. We therefore aimed to determine the physiologic, age-related MUD trend of biceps, quadriceps, tibialis anterior, hamstrings, gluteal and calf muscles, from pre- to the first year of postnatal life. To avoid a bias by pregnancy-related signal disturbances, we expressed fetal MUD as a ratio against bone ultrasound density. We used the full-term prenatal MUD ratio and the newborn postnatal MUD value as reference points, so that MUD development could be quantified from early pre- into postnatal life. Results: During the prenatal period, the total muscle group revealed a developmental MUD trend concerning a fetal increase in MUD-ratio from the 2^nd^ trimester up to the end of the 3^rd^ trimester [median increase: 27% (range 16–45), *p <* .*001*]. After birth, MUD-values increased up to the sixth month [median increase: 11% (range -7-27), *p = 0*.*025]* and stabilized thereafter. Additionally, there were also individual MUD characteristics per muscle group and developmental stage, such as relatively low MUD values of fetal hamstrings and high values of the paediatric gluteus muscles. These MUD trends are likely to concur with analogous developmentally, maturation-related alterations in the muscle water to peptide content ratios.

## Introduction

Muscle ultrasound provides a non-invasive and easily applicable tool for the detection of muscle alterations in children and adults suspected of neuromuscular pathology [1–5]. The term muscle ultrasound density (MUD), which is also indicated as echo-intensity or echogenicity, is based on the principle that a reduction in the muscle water content, fat deposition and fibrosis cause an increased reflection of the muscle ultrasound beam and thereby enhance the quantifiable MUD value (resulting in enhanced echo-intensity or echogenicity) [6–16]. Especially in fetuses and young children, this non-invasive surveillance technique has the advantage above other more invasive approaches, such as anesthesia requiring MRI and/or muscle biopsy performances [3,16]. Well-known pediatric neuromuscular applications of this technique involve for instance: neuromuscular and metabolic disorders, including spina bifida, myopathies, motor neuron diseases, neuropathies, mitochondrial disorders and glycogen storage diseases [3,5,16–19].

In healthy children older than two years of age, it has been reported that MUD values may generally remain stable [15], although there might be some exceptions in individual muscles [20]. However, in fetuses and infants younger than one year of age, healthy control values and potential age related trends are still lacking. In perspective of the pre- to postnatal developmental change in the muscle -water and -peptide content, we reasoned that MUD values could change, accordingly [21]. In healthy fetuses and infants under one year of age, we thus aimed to explore the temporal relationship between MUD trends and gestational age. Developmental insight in perinatal MUD control values could contribute to the understanding of physiologic muscle maturation, could enable cross-sectional comparison between innovative fetal treatment strategies (such as in fetal open and endoscopic closure of the neural tube defect) and also enable non-invasive, longitudinal surveillance of perinatal neuro-muscular abnormalities.

## Materials and methods

### Participants

The medical ethical committee of the University Medical Center Groningen (UMCG) approved the study. Informed consent was obtained from all parents in accordance with the World Medical Association Declaration of Helsinki 2008. After informed consent by the parents, we assessed MUD parameters in 20 healthy fetuses and infants. By open advertisement, we approached pregnant mothers of healthy fetuses who performed ultrasound (US) scans for private reasons (‘non-medical’ US recordings). All parent(s) gave their informed consent for off-line muscle ultrasound assessment of the fetal ultrasound recordings. US registrations were performed by a professional sonographer (P.B.M., not involved in image analysis). Inclusion criteria were: healthy singleton pregnancies. Exclusion criteria were: twin-pregnancies, maternal diseases, medication, congenital malformation and complications such as asphyxia, infections, cerebral bleedings and infarctions.

We included 20 fetuses. Delivery was at 40^+1^ weeks gestational age (GA) (median, range 38^+3^–41^+5^). All children were delivered after an uneventful pregnancy in absence of perinatal complications. All lengths and weights of the included fetuses and newborns were within the normal range of the prenatal Hadlock growth curve and the postnatal growth curve [22]. Since weight and length of healthy children do not influence MUD [15,20], individual values are not specified. For personal reasons related to traveling and domestic zest, parents of eight fetally included children decided to stop with their longitudinal participation after delivery. These children were “replaced” by eight healthy newborns that fulfilled the same inclusion criteria. Before processing the data of these eight neonates as part of the postnatal study group, we verified whether early neonatal MUD outcomes (at 0 months of age) statistically differed between these and the other 12 fetally included neonates. This was not the case (*NS*, *Mann-Whitney-U*).

From five perinatal autopsies in fetuses and infants without neuromuscular disease, iliopsoas muscle samples were taken at GA 21^+3^, 31^+2^, 39^+4^ (immediately after birth), 8 months postnatal and 12 months postnatal. In such autopsies iliopsoas is routinely sampled and these tissue slides were therefore present for evaluation, according to the ‘Code of Conduct for dealing responsibly with human tissue in the context of health research' published by the Federation of Dutch Medical Scientific Societies in 2011 [23].

### Muscle ultrasound assessments

Prenatally, we assessed MUD of biceps, quadriceps, tibialis anterior, hamstrings, gluteal and calf muscles at three time points: 20–24 weeks GA, 28–32 weeks and 36–40 weeks GA. Postnatally, we assessed MUD of the same muscles at 0, 6 and 12 months of age.

### Fetal muscle ultrasound assessments

Fetal muscle ultrasound recordings were performed using a *General Electric Healthcare Voluson E8* ultrasound machine. To avoid a bias by pregnancy-related signal disturbances (such as the maternal subcutaneous fat layer and/or the intra-uterine fetal conditions), we expressed fetal-MUD as a ratio between muscle- and bone- density: fetal-MUD-ratio = [mean muscle pixel value] / [mean bone pixel value] [16]. For analysis of the fetal muscle, we selected the whole muscle in a longitudinal section as the region of interest, see S1A Fig. The fetal-MUD-ratio was assessed for the biceps (reference bone: humerus), quadriceps (reference bone: femur), tibialis anterior (reference bone: tibia), hamstrings (reference bone: femur), gluteus (reference bone: hip) and calf muscles (reference bone: tibia or fibula). From each set of five images per muscle per fetus, we derived one data point by excluding the highest and lowest value and calculating the mean of the remaining three MUD values.

### Postnatal muscle ultrasound assessments

Postnatally, we obtained absolute MUD values using *General Electric Healthcare LOGIQ 9* (fixed) or *General Electric Healthcare LOGIQ e* (portable) US machines (Jiangsu, China). The registrations were performed at the outpatient clinic of Clinical Neurophysiology, or at home (using the portable device) when parents were unable to attend the appointment at the outpatient clinic. Both ultrasound machines are compatible systems, calibrated by *General Electric* technicians. We performed MU registrations with standardized settings for muscle ultrasound gain, dynamic range, compression, and time-gain compensation parameters [10,11]. All settings for muscle ultrasound assessments were identical and held constant for each machine. This setting has been shown to result in highly compatible results, with a validated conversion factor between both machines: MUD_logiq 9_ = 37.262 + 1.368 * MUD_logiq e_ (r^2^ = .74) [24].

According to standardized reference points [10], we recorded transverse US images of a total of six muscles of upper arm, thigh and leg muscles (i.e. biceps, quadriceps, tibialis anterior, hamstrings, gluteus and calf muscles). The US assessments were performed during muscle relaxation in supine (biceps, quadriceps and tibialis anterior muscles) and prone (hamstrings, gluteus and calf muscles) position, respectively. At 0, 6 and 12 months postnatal age, we assessed the children in a quiet and awake state [25]. Digital MUD values were obtained according to standardized methodology, in which images of muscle selections are made at the cross-sectional plane from the muscle belly, see S1B Fig [10,11,15]. We recorded five transverse muscle ultrasound images per muscle (of the left and right extremities in each child). From each set of five images per muscle per child, we derived one data point by excluding the highest and lowest value and calculating the mean of the remaining three MUD values. For digital pixel analysis of the fetal and postnatal muscles, we used Adobe Photoshop (San Jose, CA).

### Developmental MUD trend

Since fetal-MUD is assessed as a muscle to bone ratio [16] and postnatal MUD as an absolute value [6–15], we used the full-term prenatal MUD ratio and the newborn postnatal MUD value as a reference point. Accordingly, we set the full-term fetal-MUD-ratio value at 100% and calculated the %MUD difference between the two earlier fetal time points. Analogously, we set the newborn MUD value at the reference value of 100% and calculated the difference between the two consecutive time points at 6 and 12 months of age.

### Statistical analysis

We performed statistical analysis by SPSS version 16.0 (SPSS Inc., Chicago, IL, USA). MUD values were not-normally distributed (according to Q-Q-plots and Shapiro-Wilk tests). We compared the differences in fetal-MUD ratio between the second trimester to the end of the third trimester of pregnancy (nonparametric Kruskal-Wallis test) for the total muscle group (i.e. all investigated muscles together), and performed post hoc analysis by Mann Whitney-U test. After birth, we cross-sectionally compared MUD values for the total muscle group between 0, 6 and 12 months postnatal age by Kruskal-Wallis test and also performed post hoc analysis by the Mann-Whitney-U test. Inter-individual MUD differences (i.e. differences between muscles) were determined by the Mann-Whitney-U test. Statistical significance was set at α = .05.

## Results

### Fetal MUD-ratio

**Fig 1:**
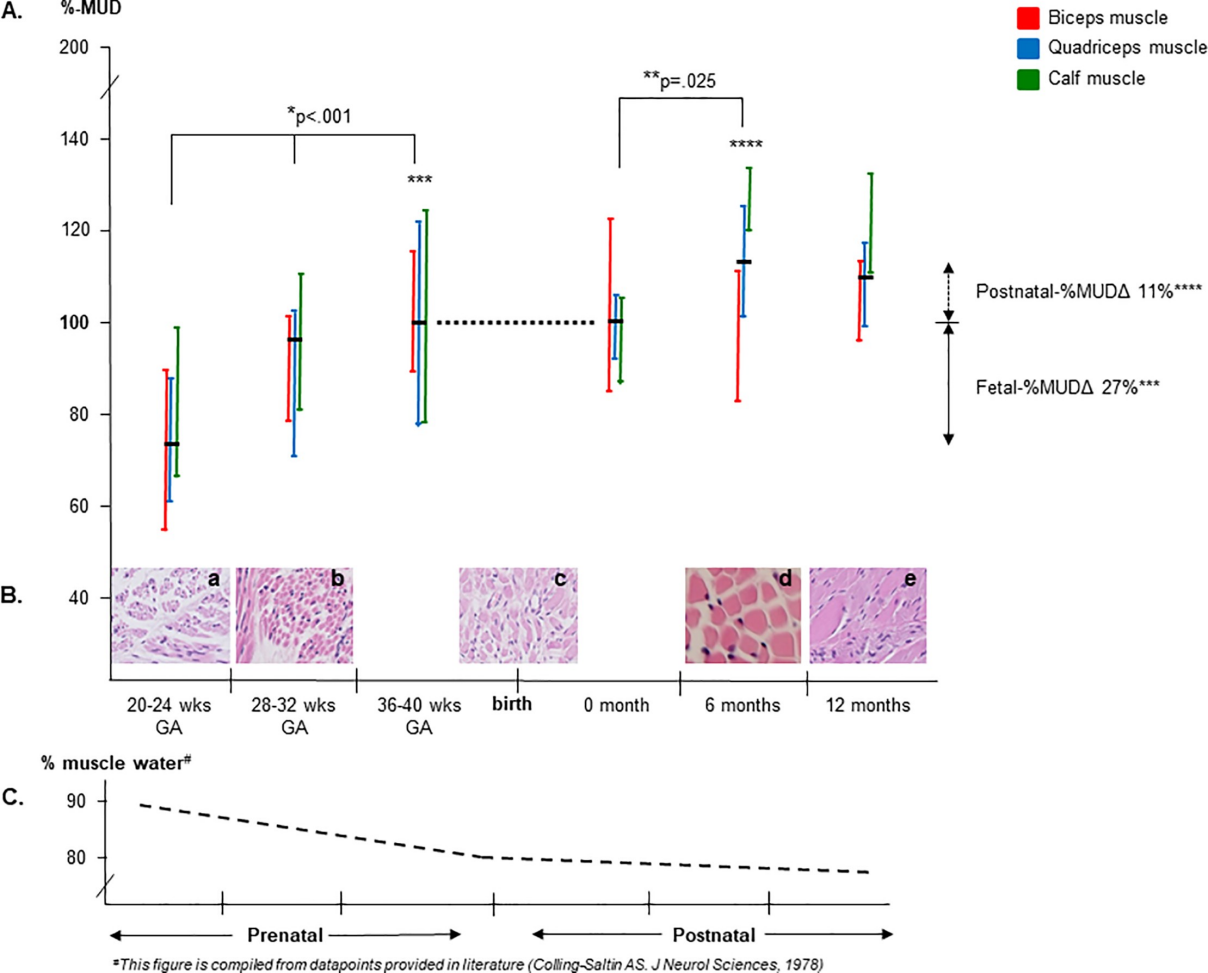
Physiologic trend of MUD in biceps, quadriceps and calf muscles in healthy fetuses and infants. Section A. Developmental pre- and postnatal MUD alterations of biceps, quadriceps and calf muscles, expressed as % change in MUD (using a-term/newborn MUD values as reference point). Fetal-MUD parameters are expressed as the ratio between muscle- and bone- density: [mean muscle pixel value] / [mean bone pixel value]. Postnatal-MUD parameters are directly obtained as the absolute muscle ultrasound density of the muscle. The a-term fetal values and the newborn postnatal values are coupled and set as a reference at 100%. From the a-term/newborn reference points, we calculated the %differences in pre- and postnatal MUD parameters from the second trimester of gestation until the first year of postnatal life. The whiskerplots indicate the medians and 1^st^ and 3^rd^ quartile (shown for biceps, quadriceps and calf muscle). In healthy fetuses, the MUD-ratio of the total muscle group increased from the second trimester (20–24 weeks) of pregnancy until the third trimester (36–40 weeks) of pregnancy (*p<0*.001*). The median %increase of fetal MUD was 27% (***). Postnatally, MUD increased from birth until the sixth month postnatal age and stabilized thereafter (*p =* .025**). The median %increase of postnatal MUD was 11% (****). Section B. Illustrations of cross-sectional muscle histology sections (hematoxylin-eosin staining; 400x) from iliopsoas muscles of healthy fetuses and infants showing how %MUD alterations may correspond with pre- and postnatal limb muscle histology. Insert a: fetal section at 21^+3^ weeks GA. Insert b: fetal section at 31^+2^ weeks GA. Insert c: neonatal section at 39^+4^ GA. Insert d: postnatal section at 8 months of age. Insert e: postnatal section at 12 months of age. All inserts are derived from the UMCG pathology database. Section B illustrates how developmental %MUD alterations of section A concur in time with developmental muscle alterations in fiber diameter and muscle fiber density. Section C. Graph compiled from the reported % muscle water content in muscle samples (expressed in percent of the total wet weight of the muscles). The biochemically determined muscle water content changed from 89% at 25 weeks of gestation to approximately 80% at delivery, to 78% at the first year of life [21]. Section C illustrates how the reported alterations in %MUD outcomes (reflecting the relative muscle water content) from section A correspond with previously reported biochemical outcome data on the muscle water content. We assembled this graph from the data previously reported by Colling-Saltin, J Neurol Sciences 1978 [21]. For the absolute values of all pre- and postnatal MUD parameters (median and ranges), see Table 1. MUD = muscle ultrasound density; GA = gestational age.

In healthy fetuses and infants, MUD values of the total muscle group (biceps, quadriceps, tibialis anterior, hamstrings, gluteal, calf muscles) increased during pregnancy (from the second trimester until late in the third trimester), Kruskal-Wallis test, *p <* .*001*. Post-hoc analysis (Mann-Whitney-U) showed the lowest MUD-ratio at 20–24 weeks GA, which increased up to 36–40 weeks GA [20–24 weeks GA: 0.22 (0.13–0.42) vs 28–32 weeks GA: 0.27 (0.14–0.41) and 36–40 weeks GA: 0.28 (0.13–0.55), respectively; *p <* .*001*]. For all muscles together, the median %fetal-MUD-ratio increase was 27% (range 16–45%), Fig 1 (data shown for biceps, quadriceps and calf muscles). The median fetal MUD-ratio values per muscle per time point are indicated in Table 1. Comparing the fetal MUD-ratio between the different muscles revealed relatively lower MUD-ratios in hamstrings muscles in the third trimester of pregnancy (Kruskal-Wallis, Post-hoc, *p <* .*05*).

**Table 1:** MUD values in healthy control fetuses and infants.

	Fetal-MUD-ratio			Postnatal-MUD		
	2nd (20–24 wks)	3rd (28–32 wks)	3rd (36–40 wks)	0 (0–2 mo)	6 (5–8 mo)	12 (11–12 mo)
Biceps	.24 (.16-.37)	.32 (.19-.41)	.33 (.23-.46)	67 (48–92)	65 (38–83)	70 (51–87)
Quadriceps	.22 (.15-.38)	.30 (.22-.37)	.32 (.19-.56)	72 (53–81)	83 (58–96)	77 (64–93)
Tibialis anterior	.25 (.17-.42)	.28 (.17-.35)	.29 (.21-.48)	75 (57–96)	70 (53–100)	70 (59–89)
Gluteus	.20 (.13-.38)	.24 (.16-.30)	.26 (.18-.38)	95 (63–124)	102 (80–157)	92 (73–126)
Hamstrings	.19 (.16-.28)	.21 (.14-.30)	.22 (.13-.29)	61 (46–76)	64 (51–86)	67 (50–85)
Calf	.25 (.16-.37)	.29 (.19-.39)	.29 (.15-.38)	71 (61–81)	90 (62–104)	84 (69–110)

Fetal-MUD is expressed as the ratio between muscle- and bone density: [mean muscle pixel value] / [mean bone muscle pixel value] and obtained at the indicated weeks of GA. Postnatal-MUD is directly obtained as the absolute muscle ultrasound density of the muscle [mean muscle pixel value] and obtained at the indicated months postnatal age. All values are indicated as median values (ranges = minimum-maximum). MUD = muscle ultrasound density; wks = weeks; mo = months.

### Postnatal MUD

Postnatally, median MUD values of the total muscle group were different between the three measurement points (Kruskal-Wallis test; *p =* .*019*). Post-hoc analysis (Mann-Whitney-U) showed a higher median MUD value at 6 months than at birth [for all muscles: 80 (38–157) vs 72 (46–124), respectively; *p =* .*025*] and a stabilization thereafter, see Fig 1. The median %increase of postnatal MUD was 11% (range -7-27%). The median MUD values per muscle and per measurement point (0, 6 and 12 months) are provided in Table 1. From Table 1 it can be derived that not all individual muscles showed the same global developmental trend in MUD. For instance, the MUD values of biceps and tibialis anterior muscles did not increase from 0 to 6 months of age. Postnatally, MUD values of the gluteus muscle were higher than that of other muscles (Kruskal-Wallis, Post-hoc, *p <* .*001*).

## Discussion

In healthy fetuses and infants until the first year of life, we investigated developmental changes in MUD. Between the 2^nd^ and 3^rd^ trimester of pregnancy, we observed an increase in fetal MUD values, continuing until the sixth month of postnatal life for the total muscle group and a relative stabilization, thereafter. These developmental MUD changes concur in time with histologic alterations and physiologic shifts in the muscle peptide and water content. Although this trend appeared globally applicable, individual muscle characteristics may still coexist. These data may implicate that perinatal MUD outcome parameters should be interpreted for gestational age and muscle type.

**Fig 2:**
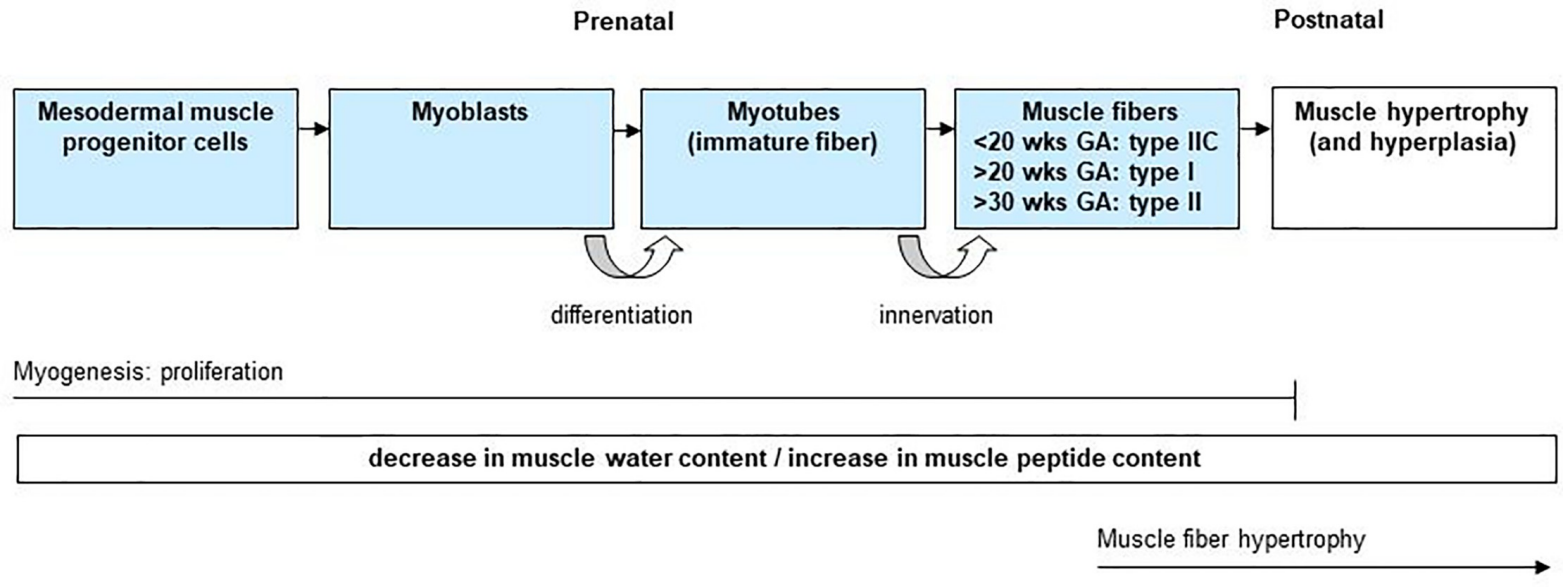
Schematic overview of physiological muscle maturation during pre- and postnatal life (until the first year after birth). During embryogenesis, muscle fibres are derived from mesodermal progenitor cells. These cells are determined to become myoblasts and differentiate into multinucleated myotubes (immature muscle fibre). Maturation of myotubes into muscle fibres is completed by innervation. At the end of gestation, myogenesis is nearly complete. Postnatally, muscle growth is mainly controlled by hypertrophy (i.e. increase in fibre diameter and length without significant change in myonuclear number) and to a very limited extent by muscle hyperplasia (i.e. increase in cell number) [27,28]. The process of muscle development is associated with an overall decrease in muscle water content and an increase in muscle peptide content [21,26,27] potentially underlying the observed age-related increase in MUD values. MUD = muscle ultrasound density.

From pre- to postnatal life, histologic muscle development is known to concur with incremental changes of the muscle peptide to water ratio [21,26], which will enhance the reflection of the muscle ultrasound beam causing increased MUD values. In Fig 2, we provide an overview of the perinatal histologic muscle development.

During embryogenesis, fetal muscles develop from the mesodermal progenitor cell [29,30]. After neuronal innervation, these precursor cells differentiate into multinucleated myotubes (i.e. immature muscles fibres) and subsequently into muscle fibres (Fig 2). During the 2^nd^ trimester of pregnancy (from 20 to 30 weeks GA), undifferentiated muscle fibres type IIC differentiate into muscle fibres type I and II [26]. By the end of pregnancy, myogenesis is nearly completed with only a small percentage (15 to 20%) of undifferentiated muscle fibres. During the newborn period, muscle development will continue with: 1. muscle fibre hypertrophy [27,28]; 2. gradual increase in muscle fibre diameter and in type I and II fibres, and 3. a relative reduction in the undifferentiated muscle fibres [26].

Comparing developmental MUD alterations before and after birth, reveals a stronger MUD increase before birth. This can be attributed to the strongest physiologic changes in the fetal muscle. During prenatal life, the muscle protein content increases from 80 milligram protein per gram muscle at 25 weeks gestational age to 155–170 milligram protein per gram muscle after the 36 weeks of gestation [21,26]. Analogously, the water content of fetal muscles (expressed as % total wet muscle weight) declines from 89% at 25 weeks of gestation to approximately 80% at delivery. During the postnatal period, the decline in the water content is smaller, i.e. 80% at birth, to 79% at the first month and to 78% at the first year of life [21].

In addition to the above described developmental MUD trends related to the maturation of the muscle, we also observed some individual differences between muscle groups. These variations can be theoretically attributed to: 1. differences in muscle fibre type differentiation per muscle [29–31]; 2. differences in the genetic profile and/or DNA content between the muscles [27,29,32]; 3. differences in ‘slow twitch’ (for endurance, type I) and ‘fast twitch’ characteristics (for strength, type II) and 4. differences between the distribution of fibre types over the muscle (depending on the muscle function and the influence by activity, muscle force and exercise) [27,33]. However, regarding the considerable ranges in MUD outcomes, one should be reluctant with strong (over)interpretations of individual outcomes.

Altogether, in fetuses and young infants under one year of age, MUD values of human muscles reveal a physiological developmental increase with age, despite potentially inter-individual differences and specific muscle characteristics per muscle group. These data may implicate that non-invasive MUD assessment can be diagnostically used [3,5,16,19,34], however, on the condition that outcome data are interpreted for gestational age.

We recognize some weak points to this study. First, to avoid confounding influences (by the maternal sub-cutaneous fat layer and the fetal intra-uterine position), we had to express fetal-MUD by the muscle to bone-ratio, instead of by the absolute value. However, in previous studies we have shown that MUD-ratios can be used for prenatal surveillance purposes, as well [16]. Furthermore, by setting a-term age as a reference point, longitudinal trends can be reliably derived. Second, like any biological sample or parameter, we are aware that there are inter-individual differences such as for the fetal muscle glycogen content [21], subcutaneous fat layers [10,11,15], muscle size and body mass index [15] and, finally, there may be consequences by technical, measurement related factors [10,35]. However, despite these limitations, we observed an identical developmental MUD trend for all investigated fetal muscles that can be attributed to ongoing muscle maturation. Postnatally, this developmental trend continued, but attenuated in the total muscle group. Additionally, we noticed moderate deviations from the total MUD trend in some individual muscles (biceps and tibialis anterior muscles). Although we are reluctant to over-interpret these findings, it is tempting to hypothesize that these muscles could still reveal the same developmental trend, after a short delay. For instance, in this respect Lori et al. had reported that biceps and tibialis anterior muscles do reveal an MUD increase, after the 2^nd^ year of life [20]. However, additional data are needed to substantiate this hypothesis to further extent.

## Conclusions

In healthy fetuses and infants, limb muscles revealed a trend with increasing MUD values from prenatal life to 6 months of age, followed by a relative stabilization, thereafter. In fetuses and children younger than one year of age, these data may implicate that MUD outcome parameters of limb muscles should be interpreted for gestational age. These findings may provide a non-invasive tool for perinatal neuromuscular surveillance, potentially allowing quantitative comparison between different innovative fetal and neonatal treatment strategies.

## Supporting information

S1 Fig Ultrasound images of the fetal and postnatal quadriceps muscle. a. Fetal ultrasound image of the quadriceps muscle. The image shows a longitudinal section of the quadriceps muscle. The blue encircled area indicates the region of interest for fetal muscle ultrasound analysis. b. Postnatal ultrasound image of the quadriceps muscle. The image shows a cross-section of the quadriceps muscle taken at the muscle belly of the rectus femoris. The blue encircled area (rectus femoris and vastus intermedius) indicates the region of interest for postnatal muscle ultrasound analysis.
(TIF)

S1 File (SAV)
