# Supplementary figures and images for: Development of muscle ultrasound density in healthy fetuses and infants
Source: PLoS One. 2020 Jul 10;15(7):e0235836. doi: 10.1371/journal.pone.0235836 (PMC7351181; doi:10.1371/journal.pone.0235836)

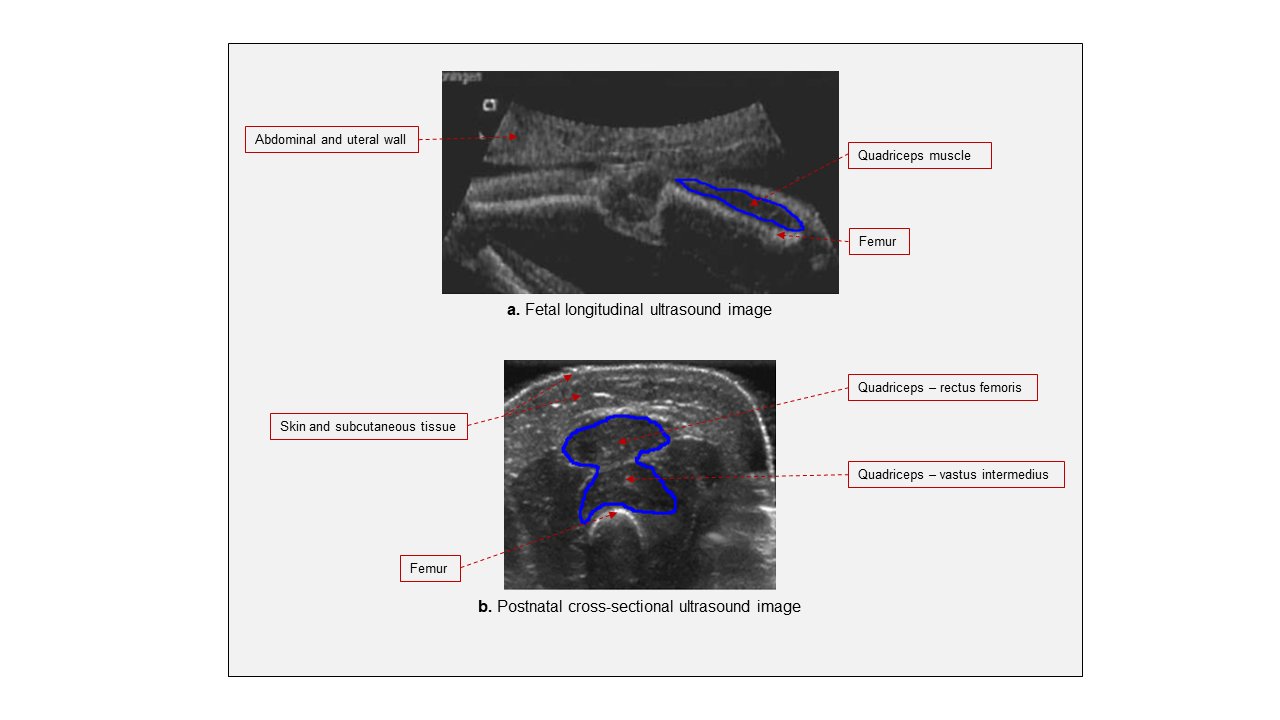

Supplement: S1 Fig — a. Fetal ultrasound image of the quadriceps muscle. The image shows a longitudinal section of the quadriceps muscle. The blue encircled area indicates the region of interest for fetal muscle ultrasound analysis. b. Postnatal ultrasound image of the quadriceps muscle. The image shows a cross-section of the quadriceps muscle taken at the muscle belly of the rectus femoris. The blue encircled area (rectus femoris and vastus intermedius) indicates the region of interest for postnatal muscle ultrasound analysis. (TIF) [file pone.0235836.s001.tif]
